# Lycopene-Loaded Emulsions: Chitosan Versus Non-Ionic Surfactants as Stabilizers

**DOI:** 10.3390/molecules29215209

**Published:** 2024-11-04

**Authors:** Sonia Álvarez-García, Lucie Couarraze, María Matos, Gemma Gutiérrez

**Affiliations:** 1 Department of Chemical and Environmental Engineering, University of Oviedo, Julián Clavería 8, 33006 Oviedo, Spain; alvarezsonia@uniovi.es (S.Á.-G.); lucie.couarraze@gmail.com (L.C.); matosmaria@uniovi.es (M.M.); 2 ENSMAC—Bordeaux INP, 16 Avenue Pey Berland, 33600 Pessac, France; 3 Instituto Universitario de Biotecnología de Asturias, University of Oviedo, 33006 Oviedo, Spain

**Keywords:** lycopene, chitosan, emulsions, encapsulation, stability, sustainable

## Abstract

Lycopene is a natural carotenoid with well-known benefits due to its antioxidant properties, including an anti-inflammatory effect in colorectal cancer and anti-angiogenic effects along with a reduction in the risk of prostate cancer and coronary heart disease. Due to their poor water solubility, photosensitivity and heat sensitivity, their incorporation in cosmetic and food matrices should be through encapsulation systems. In the present work, lycopene-loaded emulsions were prepared using two different types of stabilizers: non-ionic surfactants, testing several ratios of Tween 80 and Span 80, and chitosan, using chitosans of different viscosities and molecular weights. Soybean oil was found to be a suitable candidate for O/W emulsion preparation. Lycopene encapsulation efficiency (EE) of 70–75% and loading capacities of 0.14 mg/g were registered in stable emulsions stabilized either by non-ionic surfactants or acidified chitosans. Therefore, chitosan is a good alternative as a sustainable stabilizer to partially replace traditional synthetic ingredients with a new biodegradable, renewable and biocompatible material which could contribute to reduce the environmental impact as well as the ingestion of synthetic toxic materials by humans, decreasing their risk of suffering from chronic and complex pathologies, among which several types of cancer stand out.

## 1. Introduction

Lycopene, an acyclic pigment, is a member of the carotenoid family, specifically belonging to the carotene subgroup [1]. This compound is predominantly located in the chromoplasts of red fruits and vegetables, notably tomatoes, peppers, watermelons and grapefruits [2]. Owing to its hydrophobic characteristics, lycopene exhibits poor solubility in water and alcohol, yet it dissolves well in oils and organic solvents such as acetone, hexane and chloroform [3].

Lycopene is a biocompound of interest due to its antioxidant properties, capable of neutralizing reactive oxygen species (ROS) and reactive nitrogen species (RNS) that are generated during oxidative stress [4]. It has been associated with numerous health benefits, including an anti-inflammatory effect in colorectal cancer [5], anti-angiogenesis [6] and a reduction in the risk of prostate cancer [7] and coronary heart disease [8]. Consequently, it holds potential as a functional food ingredient. However, the photosensitive and heat-sensitive nature of lycopene limits its therapeutic applications. To protect lycopene and maintain its properties over time, encapsulation is necessary. On the other hand, the bioavailability of lycopene is inherently limited and is contingent upon a multitude of factors, inclusive of its co-ingestion with oil [9]. Between the different technologies available for lycopene encapsulation, emulsions have been revealed as a successful strategy [10,11,12]. An emulsion is a system formed by two phases, where one disperses as drops in the other and the drops are suspended on the external phase and stabilized using amphiphilic molecules such as surfactants, proteins or solid particles (of organic or inorganic nature) with a capacity to be adsorbed on the oil/water interface. The characteristics of particles (wettability, size, concentration, shape, surface charge, etc.) have multiple effects on Pickering emulsions, such as stability, drop size, size distribution and other characteristics (rheology, encapsulation properties among others) [13].

In this work, oil-in-water (O/W) emulsions were synthesized for lycopene encapsulation for their further incorporation into a food matrix. In oil-in-water emulsions, oil droplets are dispersed within an aqueous continuous phase.

Numerous studies in the literature have reported the encapsulation of lycopene in simple O/W emulsions using various types of emulsifiers, including the following: (i) synthetic surfactants such as Tween 20 [10], SDS [11] and Brij 10 [14], (ii) proteins like pea protein isolate [12] and sodium caseinate [15] and (iii) other macromolecules such as starches (e.g., rice, corn, quinoa, etc.) [16,17]. Additionally, encapsulation of lycopene in O/W emulsions has been performed using different types of oils such as corn oil [18], linseed oil [19], sesame oil [20] and MCT [21], among others.

In the current investigation, encapsulation is performed in simple O/W emulsions. The novelty of this work lies in two aspects: the use of a natural oil (soybean) in the formulation and the employment of two different types of stabilizers: non-ionic surfactants and chitosan. Chitosan is the second most abundant biopolymer in nature and shows good properties in terms of sustainability, circularity and energy consumption during industrial applications [20]. Conventional nanoformulations have been predominantly controlled by synthetic or low-molecular-weight substances on account of their affordability and simplistic application [21]. Consumers may be exposed to carcinogenic substances and allergy-like reactions due to this convenience [22]. The idea is to replace the traditional synthetic ingredients by new biodegradable, renewable and biocompatible materials which could contribute to reduce both (i) the environmental impact and (ii) the ingestion of synthetic toxic materials by humans, reducing their risk of suffering from chronic and complex pathologies, among which several types of cancer stand out.

The two non-ionic surfactants used are Tween 80 (polyoxyethylene sorbitan monooleate) and Span 80 (sorbitan monooleate), which are both synthetic non-ionic surfactants with different behavior due to their molecular structure. Thus, the presence of POE chains in Tween 80 makes it water-soluble and hydrophilic (HLB= 15), whereas Span 80 is lipophilic and soluble in oils and organic solvents (HLB = 4.3) [23]. On the other hand, chitosan is a polysaccharide obtained after deacetylation of the chitin extracted from crustaceans that can act as stabilizer [24]. Its antimicrobial [25] and antibacterial properties and biocompatibility [26] make it an interesting compound to use in food formulations. In the present work, five different types of chitosan have been used with different molecular weights and viscosity. The present work allows us to compare in terms of stability, drop size and encapsulation efficiency (EE) of O/W emulsions stabilized by traditional surfactants and the use of sustainable alternatives such as chitosan.

Natural oils have promising properties that could increase, or even produce a synergic effect with, the benefits provided by lycopene. The natural oil used in this work is soybean oil, which is the world’s leading vegetable oil for edible consumption and is often used in the field of emulsions. 

Soybean oil is rich in heart-healthy fatty acids (omega-3 and omega-6), which are beneficial for the health of the skin, bones and the coronary system by lowering cholesterol [27,28].

In this work, the antioxidant compound lycopene was encapsulated in stable O/W emulsions using sustainable formulations. To do this, it was necessary to optimize the emulsion formulation, studying the effect of the type of oil used, as well as the type of stabilizers and its combinations and ratios. Prepared emulsions were characterized in terms of stability, droplet size, morphology and rheological behavior. Lycopene was encapsulated in selected O/W emulsions, obtaining systems that increase the chemical stability of lycopene during storage, and the EE was evaluated in both types of emulsions prepared (surfactants or chitosan stabilized emulsions). 

## 2. Results and Discussion

Emulsions prepared with different emulsifiers were characterized in terms of size, morphology and stability.

### 2.1. Effect of Types of Emulsifiers on the Particle Size and Particle Size Distribution

- Emulsions stabilized with surfactants

**Figure 1:**
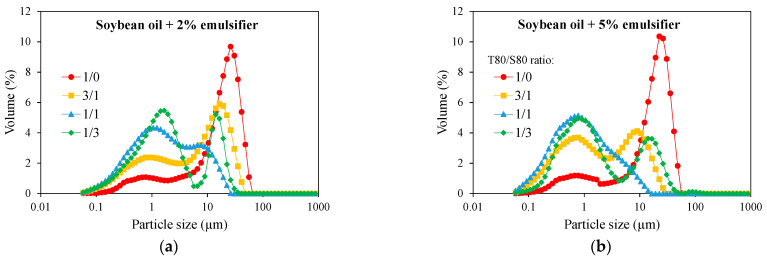
Particle size distribution of emulsions prepared with soybean oil and 2% (**a**) or 5% (**b**) of emulsifier at different ratios of Tween 80/Span 80.

Figure 1 shows the droplet size distributions of the emulsions formed using different percentages and ratios of T80 and S80 as stabilizers. As can be observed, the droplet size slightly decreases when the total percentage of emulsifier increases. That means that the more surfactant is used, the more the surface tension decreases, allowing the formation of more oil/water interface area and hence reducing droplet size.

The PSD of all emulsions are polydisperse, showing two main peaks: a first peak, corresponding to particles ~2 µm and a second peak that corresponds to particles between 10 and 26 µm. The high viscosity of soybean oil (50.09 mPa·s at 25 °C) means that high shear is necessary to create an interface [29], enhancing the existence of several droplet size populations. 

On the other hand, it is evident that the type of emulsifier used influences the droplet size. Thus, when only T80 is used as emulsifier, regardless of the percentage used, the higher droplet sizes are obtained (samples with T80/S80 = 1/0 provided droplets of ~20–25µm). The addition of S80 to the emulsion causes a decrease in droplet size. This is due to the lower solubility of S80 in water that reduces the surfactant curvature, reducing droplet size in turn [30].

- Emulsions stabilized with chitosans

**Figure 2:**
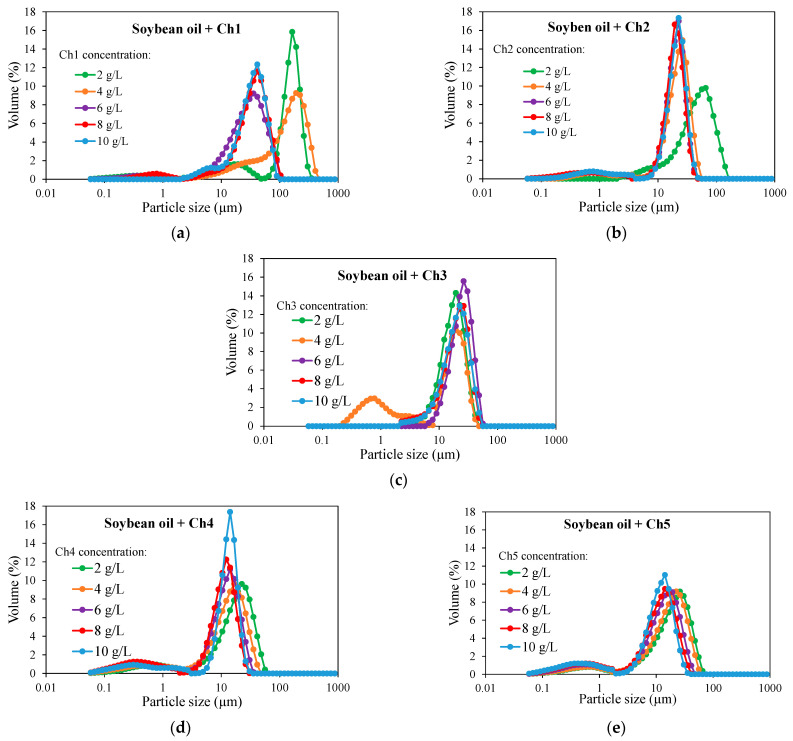
Particle size distributions of emulsions prepared with soybean oil and several concentrations of chitosans with different viscosities: 5–20 mPa·s (**a**), 20–100 mPa·s (**b**), 200–600 mPa·s (**c**) and different molecular weight: 100–300 kg/mol (**d**), 600–800 kg/mol (**e**).

**Figure 3:**
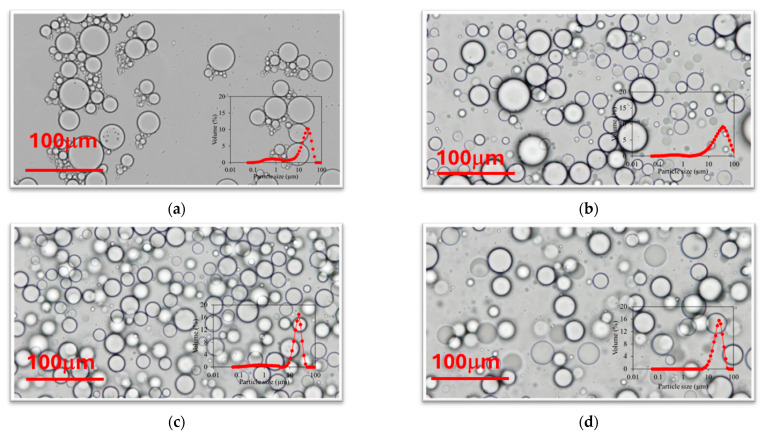

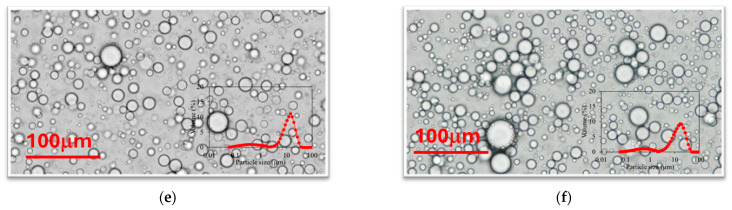
Optical microscopy images and PSD distribution (inside) of oil-in-water emulsions prepared with soybean oil and (**a**) 5% T80/S80 = 1/0, (**b**) 6 g/L of chitosan 5–20 mPa·s, (**c**) 6 g/L of chitosan 20–100 mPa·s, (**d**) 6 g/L of chitosan 200–600 mPa·s, (**e**) 6 g/L of chitosan 100–300 kg/mol and (**f**) 6 g/L chitosan 600–800 kg/mol.

As was aforementioned, two different types of chitosan were used; one type was acidified with citric acid while the other type was just pellet of chitosan. Figure 2 and Figure 3 show the PSD of the emulsion formed using different types and concentrations of chitosan.

In general terms, and contrary to what happened when using T80/S80, it can be seen that the PSD of emulsions with chitosan have unimodal distributions (in some particular cases can be bimodal, but one of the particles sizes is highly favored over the other). Polydispersity depends on the type and concentration of chitosan. 

Focusing on emulsions stabilized with acidified chitosan, it can be observed that emulsions with Ch1 had mainly one or two types of population, depending on the concentration (Figure 2a). Using low concentrations of Ch1 (2 and 4 g/L), a bimodal distribution can be observed, favoring the peak located around 160–190 µm, while at higher concentrations (6–10 g/L), particles with a mean diameter at around 30 µm could be appreciated. However, observing emulsions with Ch1 at higher concentrations (6 and 8 g/L), emulsions with a single peak at around 30–40 µm were obtained, indicating that the higher concentration allows for obtaining a larger interfacial area.

When the viscosity of the chitosan increases, as happens with Ch2 (Figure 2b), low chitosan concentrations (2 g/L) produce large drop sizes (65 µm), while chitosan concentrations between 4 and 10 g/L produce emulsions with a single narrow peak (drop size ~20 µm). For emulsions stabilized with a chitosan of higher viscosity, Ch3 (Figure 2c), all concentrations used produce emulsions with a single narrow peak at 20–25 µm, indicating that chitosan with higher viscosity presents higher affinity for the soybean oil/water interfaces.

In the case of emulsions stabilized with chitosans of different molecular weight, Ch4 and Ch5 (Figure 2d,e), all emulsions were mainly unimodal with a peak at ~20 µm but presenting a small secondary peak at ~0.5 µm. No differences were observed for the two chitosans with different molecular weight used. However, the higher concentrations of chitosan reduced the main size of the emulsion drop for both types of chitosans used; a narrower distribution was observed with Ch4 at high concentrations. For all concentrations of Ch4 and Ch5 tested, a secondary small peak at ~0.5 µm was observed, probably due to the presence of chitosan aggregates.

Comparing the droplet sizes of the emulsions with chitosan and with T80/S80, it seems that drops are mostly larger with chitosan. For example, a range of 4–20 µm was observed with T80/S80, while a range of 13–28 µm was measured when chitosan was used. Similar results were obtained in previous works where the use of surfactant molecules or starch granules were compared as stabilizers [31].

The results observed in Figure 1 and Figure 2 are corroborated by the morphology observed with optical microscopy (Figure 3). It can be easily noted that not large differences were observed between the use of surfactants or chitosan molecules as stabilizers, specially when Ch4 and Ch5 are used. It can be observed that acidified chitosans (Ch1, Ch2 and Ch3) offer a larger drop size with high uniformity, while non-acidified chitosans (Ch4 and Ch5) offers smaller mean droplet size with larger polydispersity.

### 2.2. Effect of the Type of Emulsifier on the Stability of Emulsions with Different Oils

According to PSD and apparent emulsion stability versus phase separation, 18 samples were selected for stability analysis using the Turbiscan for 30 days at 30 °C. Droplet size, backscattering profiles (BS) and the TSI of these emulsions were analyzed after this period of time. Selected samples are collected in Table 1. 

Regarding emulsions stabilized with surfactants, high HLB T80/S80 combinations selected were 1/0 and 3/1 due to the better capacity to stabilize this type of emulsion. In the case of emulsions stabilized by chitosan, the concentration selected in all cases was 6 g/L, since this was the minimum concentration that led to full coverage in all cases.

**Figure 4:**
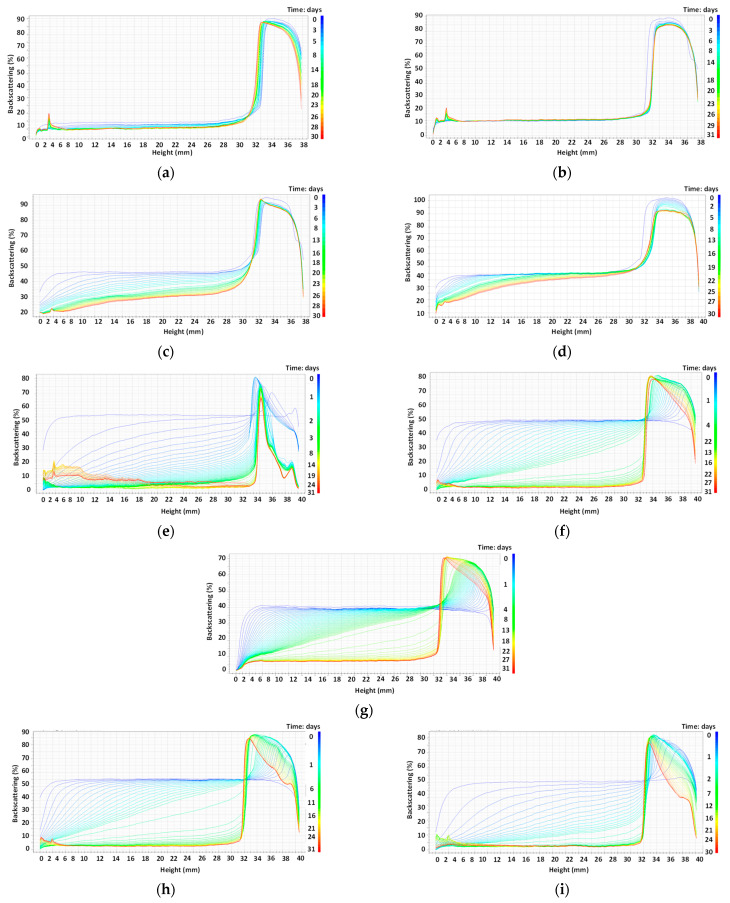
Backscattering profiles of O/W emulsions prepared with soybean oil using (**a**) 2% T80/S80, ratio 1/0; (**b**) 5% T80/S80, ratio 1/0; (**c**) 3% T80/S80, ratio 3/1; (**d**) 5% T80/S80, ratio 3/1; (**e**) 6 g/L of chitosan 5–20 mPa·s; (**f**) 6 g/L of chitosan 20–100 mPa·s; (**g**) 6 g/L of chitosan 200–600 mPa·s; (**h**) 6 g/L of chitosan 100–300 kg/mol; (**i**) 6 g/L chitosan 600–800 kg/mol. The gradient of colors represents several analyses at different periods of time, from initial time (dark blue) to 30 days (red).

BS profiles of oil-in-water emulsions prepared with T80/S80 and 6 g/L of different types of chitosan are shown in Figure 4. Initially, fresh emulsions prepared with chitosan were homogeneous, presenting as a one-layer system. However, emulsions prepared with surfactants present in all cases a creaming phenomenon from the beginning of the monitoring process. In any case, after the 30 days of analysis, it could be easily appreciated that creaming is observed in all type of emulsions. This phenomenon is due to the gravitational instability which happens when the density of the disperse phase is lower than the density of the continuous phase, causing the progressive migration of droplets upwards. These droplets create a creaming layer located at the top of the sample, which is reversible since a homogeneous phase can be obtained by gentle manual mixing. This behavior has been observed in previous studies where the stability of food-grade emulsions was monitored with time by using Turbiscan Lab (Formulaction) [32]. In polydisperse emulsions, the largest-size droplets will move upwards quickly, whereas smaller ones will remain dispersed in the lower part, according to the Stokes’s law equation. This bottom layer (rich in small oil droplets) is named the serum layer.

The difference observed at fresh conditions between the two different type of stabilizers (surfactant or chitosan) could be due to the fact that the emulsions stabilized with chitosan present higher viscosity, hindering the oil drops from rising to the surface. The size of the creaming layer will evolve with time as the drops migrate [33].

Analyzing the data obtained with the different concentrations of surfactants and different ratios between T80/S80, it can be observed that the creaming phenomena is retarded when the ratio T80/S80 3/1 was used for both surfactant concentrations, indicating that the presence of a surfactant with lower HLB acting as a costabilizer favors emulsion stability.

In the case of emulsions stabilized with chitosan, it can be observed that Ch1 (Figure 4e) present a phase separation at the top part of the emulsions, indicating the presence of a thin layer of free oil identified by the backscattering reduction on the top part. This behavior is also observed for non-acidified chitosans (Ch4 and Ch5, Figure 4h,i) but is less noticeable, being even less important in the case of the use of Ch4.

**Figure 5:**
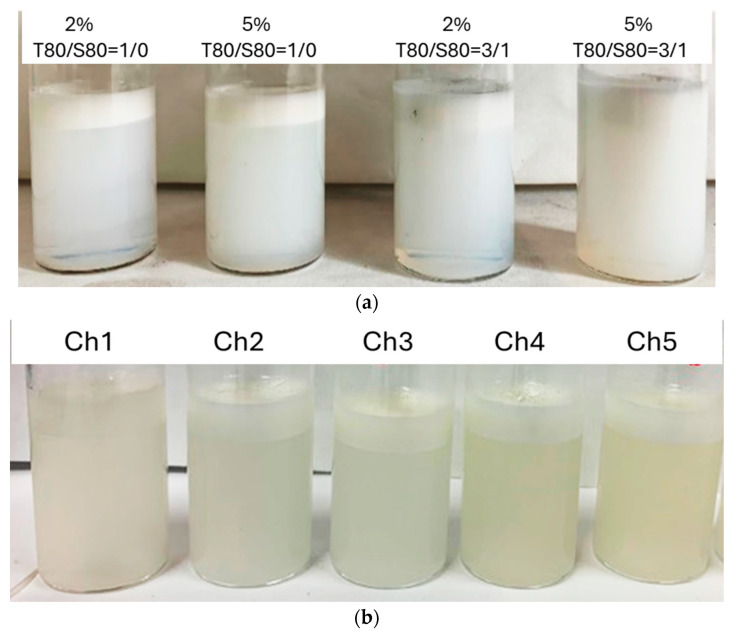
Aspect of different emulsions after 30 days, prepared with soybean oil and (**a**) different concentration of T80/S80 and (**b**) different chitosans.

Figure 5 shows photographs of the emulsions after 30 days, where several layers are observed due to the phase separation, migration and repartition of droplets in the sample. The layer in the top corresponds to the creaming layer, which can be observed for all emulsions. Below this layer there is a cloudy area, which becomes more transparent the more droplets have migrated to the surface. The bottom layer is nearly translucid because there are not enough droplets to scatter light.

In emulsions prepared with T80/S80, the serum layer is much more evident when using 2% surfactants than when using 5%. Thus, increasing the total quantity of emulsifier seems to hinder the migration of small droplets. For their part, emulsions prepared using chitosan, after one month, also present a creaming layer and a clear serum layer.

After 30 days, the particle sizes of the emulsions were measured again and the variations in particle size are shown in the Table 2. The droplets of emulsions with chitosan became bigger after 30 days. This can be the result of several instability phenomena such as coalescence and Ostwald ripening [34]. In Ostwald ripening, there is a mass transfer from the small droplets, through the continuous phase, to the larger drops, which takes place without any contact between the drops, unlike coalescence. Thus, small droplets shrink, while the largest ones grow. Ostwald ripening only occurs if the dispersed phase (oily phase) is partially soluble in the continuous phase (aqueous phase). This phenomenon has already been observed in other studies using essential oils [35]. However, when the emulsions are made using Tween80/Span80, the sizes decreased a little with time. Another phenomenon also happens due to coalescence: the larger droplets, created by the merge of smaller ones, go to the top of the emulsion, creating a thin layer of free oil. Consequently, only the small droplets remain in the emulsion.

Considering all the previous issues, we consider that soybean oil gives rise to emulsions suitable for encapsulating lycopene, and among these, we chose those that had the smaller TSI indices and variations in droplet size. Three different types of emulsions were chosen, allowing for testing of different type of stabilizers, surfactants, acidified chitosan and plain chitosan (5% of Tween80/Span80 in ratio 3/1; 6 g/L of Ch3; 6 g/L of Ch4).

### 2.3. Effect of the Type of Emulsifier in Encapsulation of Lycopene

Lycopene was encapsulated with a concentration of 0.2 g/L in the three types of emulsion (listed in Table 2).

**Figure 6:**
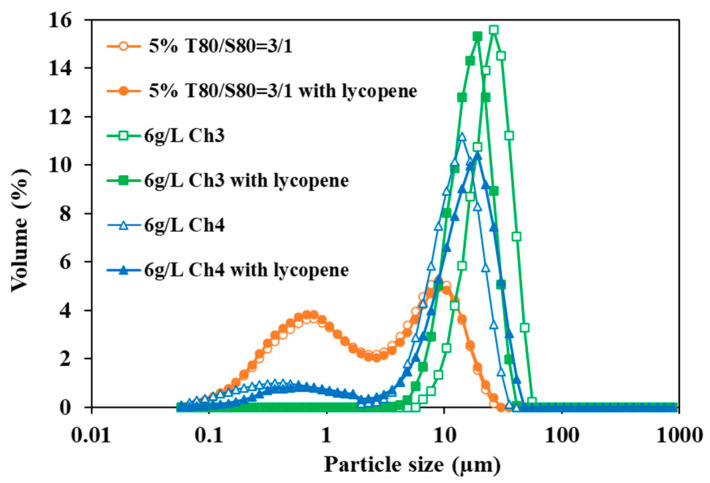
Comparison of the PSD for fresh emulsion prepared with (filled symbols) and without lycopene (hollow symbols).

Figure 6 shows the particle size distribution for the three fresh selected emulsions prepared with and without lycopene. For each type of emulsion, the curve shape is similar, regardless of whether the emulsion contains lycopene or not. Using T80/S80, the droplet size does not change when introducing lycopene, while, when using chitosan, it changes slightly (it increases from 14 to 19 µm when using Ch4 and decreases from 26 to 20 µm with Ch3). This might be due to a different interaction between lycopene and chitosan producing partial location of the lycopene at the interface. Similar results were obtained when resveratrol was encapsulated in emulsions stabilized by starch granules [31,36].

**Figure 7:**
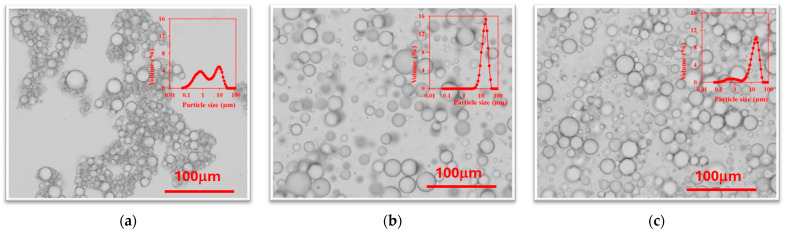
Optical microscopy images and PSD distribution (inside) of emulsions with lycopene prepared using soybean oil and (**a**) 5% T80/S80 = 3/1; (**b**) 6 g/L of chitosan 200–600 mPa·s; (**c**) 6 g/L of chitosan 100–300 kg/mol.

Optical microscopy images of the three fresh emulsions are showed in Figure 7. It can be easily observed that emulsions prepared with surfactants present a smaller drop size, and emulsions prepared with Ch4 present a larger particle size.

When emulsions are stored at room temperature and without protection from light, great destabilization is observed in just 10 days: the orange color of the emulsion phase disappears, and both a cream layer and superficial free oil layer appear.

Hence, the stability of these emulsions in the absence of light, under two different storage conditions, room temperature and 5 °C, was studied. All fresh emulsions with lycopene have just one homogeneous orange phase, whose color is more intense when chitosan is used, which is characteristic of lycopene carotenoids [37]. On the contrary, a lighter color was obtained in emulsions with surfactant as the stabilizer.

When emulsions are protected from the light, the colors remain more vibrant both at 5 °C and at room temperature. In fact, for both temperatures studied, the emulsions remain stable for the first 10 days, starting to cream after this period.

The particle sizes of fresh emulsions and emulsions stored for 10 days in the absence of light and at the two temperatures studied were measured. The variation in the D_[4;3]_ was measured for both temperatures and indicated in Table 2.

**Figure 8:**
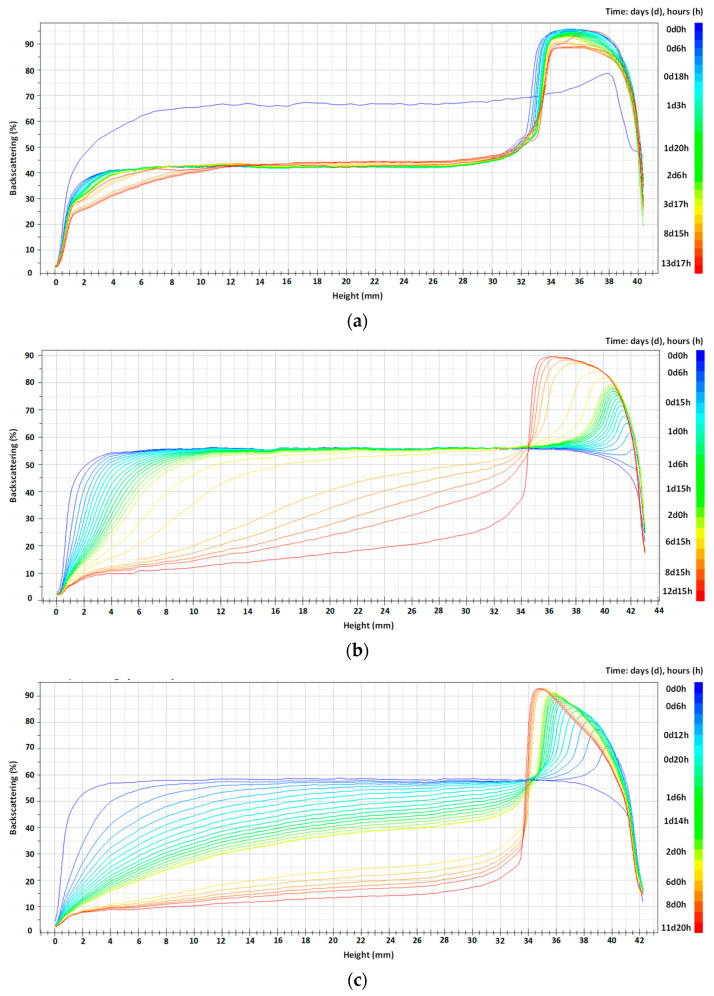
Backscattering profiles of soybean oil emulsion with lycopene, using (**a**) 5% T80/S80, ratio 3/1; (**b**) 6 g/L of chitosan 200–600 mPa·s and (**c**) 6 g/L of chitosan 100–300 kg/mol. The gradient of colors represents several analyses at different periods of time, from initial time (dark blue) to 12 days (red).

The stability of emulsions protected from light and stored at room temperature was also studied. TSI values are registered in Table 2 and backscattering profiles presented in Figure 8.

**Figure 9:**
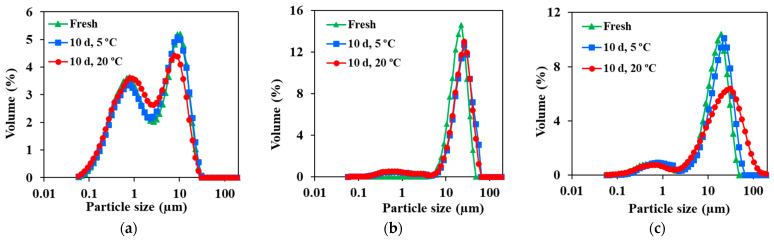
Variation of PSD during storage at 5 °C and 25 °C in absence of light for lycopene emulsions prepared with (**a**) 5% T80/S80 ratio 3/1, (**b**) 6 g/L of chitosan 200–600 mPa·s and (**c**) 6 g/L of chitosan 100–300 kg/mol.

Regarding the TSI value, the emulsion with Ch4 is more unstable after 10 days in the Turbiscan, while the other two emulsions show similar TSI values. Figure 9 shows that, after 10 days, droplet sizes have slightly increased, but not large differences were registered between fresh emulsions and after 10 days of storage (for any of the temperatures used). In the case of the emulsions stabilized with Ch3, a slight increase in droplet size was observed after 10 days for both temperature of storage (without the storage temperature appearing to affect this size increment). On the other hand, in the case of Ch4, the increase in droplet size was only obtained for samples stored at 25 °C, being hence the only case in which storage temperature has a significant influence.

Backscattering profiles show similar behavior for the three emulsions studied. A top layer of similar height can be observed for all cases. However, for surfactants used as stabilizers (Figure 8a), it seems that this layer appears from the first hours, while for the cases in which chitosan was used, a more gradual creaming could be observed. For emulsions stabilized with acidified chitosan (Ch3), the appearance of the creaming layer takes longer, indicating greater stability, which agrees with the measured TSI value.

**Figure 10:**
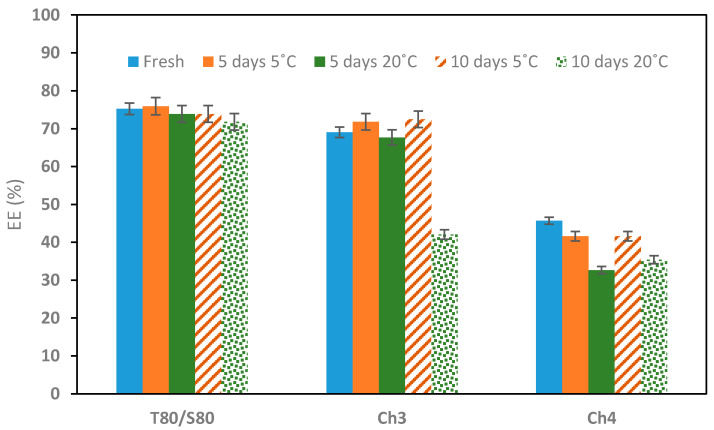
Lycopene encapsulation efficiency over time for emulsions stabilized with surfactants Tween80/Span80 at a ratio 3/1, Ch3 and Ch4.

The EE of the lycopene was registered in Figure 10. EE was measured at fresh conditions, and after 5 and 10 days of storage at 5 °C and 20 °C. Table 2 presents the loading capacity of fresh emulsions and the lycopene stability over time. Although the EE values obtained for the emulsion prepared with Tween80/Span80 are higher (EE ~75%), they are comparable with those obtained for emulsion with Ch3 (EE ~69%). However, the EE of lycopene using Ch4 is quite lower (EE ~48%). These EE values are similar to the range of values found in other studies that used different oils and surfactants to encapsulate lycopene in different types of emulsions [38,39].

## 3. Materials and Methods

### 3.1. Materials

Soybean oil is a classic oil and was provided by Sigma-Aldrich (St. Louis, MA, USA). Tween 80 (polyoxyethylene sorbitan monooleate, T80) and Span 80 (sorbitan monooleate, S80) were purchased from Sigma-Aldrich (USA). Chitosans of different viscosity and molecular weight were provided by TCI Chemical (Tokyo, Japan) (Ch1, Ch2 and Ch3) by and by Thermo Scientific (Waltham, MA, USA) (Ch4 and Ch5). Their main characteristics are summarized in Table 3. Lycopene (secondary pharmaceutical standard) was purchased from Sigma-Aldrich (USA). All chemical reagents were of analytical grade.

### 3.2. Emulsion Formulation and Preparation

Different emulsions were prepared with soybean oil, varying the type of emulsifier used and its concentration. When Tween 80 and Span 80 were used, the total concentration used was 2–5%, varying the T80/S80 proportion. On the other hand, when emulsions were prepared with the five different chitosans, their concentration was between 2 and 10 g/L.

In all cases, 30 g of the emulsions with 10% wt of oil were prepared using the rotor-stator homogenizer “Silent Crusher M” (Heidolph, Schwabach, Germany) at 15,000 rpm for 5 min.

- Emulsions with Tween 80 and Span 80

In order to prepare the aqueous phase of emulsions, the emulsifier Tween 80 was dissolved in distilled water with 0.1 M NaCl using a magnetic stirrer (500 rpm, 1.5 h, room temperature). Solutions with the desired concentrations of Tween 80 were prepared. NaCl was added as an electrolyte to decrease the difference between dielectric constants of both phases, aqueous and oily [40].

The emulsions’ oily phases were prepared by dissolving the desired amount of Span 80 in soybean oil and stirring for 1 h at 500 rpm.

In all cases, the total amount of surfactant was kept as 2 or 5%wt while varying the ratio between both surfactants (Tween 80/Span 80 ratio: 1/0, 3/1, 1/1, 1/3).

- Emulsions with chitosan

Aqueous phases of these emulsions were prepared with chitosan of different viscosity (Ch1, Ch2 and Ch3) and different molecular weight (Ch4 and Ch5). Each type of chitosan was dissolved in several concentrations (2 g/L, 4 g/L, 6 g/L, 8 g/L, 10 g/L) in acidified water solutions, since chitosan is not soluble in water at neutral pH. If the pH of the medium is lower than pKa (6.5), the amino groups of chitosan, which are sensitives to pH changes, become protonated and it is dissolved [41]. Based on this, 100 mL of distilled water was acidified with acetic acid to adjust to a pH of 3.5 and different amount of chitosan were dissolved in it to achieve the desired concentrations. A few drops of acetic acid were added until there were no more chitosan particles and the solution became transparent [42].

For these emulsions, the organic phases were constituted by plain soybean oil emulsifier.

- Lycopene encapsulation

Optimum formulations in terms of drop size and stability were selected to encapsulated lycopene at a concentration of 0.2 g/L.

Lycopene was, in all cases, previously dissolved in oil by stirring at 500 rpm during 48 h at room temperature. The oily solution was preserved from light using foil paper to avoid lycopene oxidation. For the same reason, lycopene-loaded emulsions were protected with foil paper and stored at 20 °C or 5 °C.

### 3.3. Emulsion Characterization

Prepared emulsions were characterized in terms of size, size distribution, morphology, stability and encapsulation efficiency.

- Droplet size

To determine the droplet size distribution, a Mastersizer 3000 laser particle size analyzer (Malvern Instruments Ltd., Malvern, UK) was used. All measurements were made in triplicate for each sample. The volume-averaged diameter was reported as D_[4;3]_.

Droplet morphology was observed using an Olympus BX61 light microscope (Olympus, Tokyo, Japan) with a visible source lamp.

- Emulsion stability

The stability was evaluated with Static Multiple Light Scattering (MLS) using a Turbiscan LabExpert (Formulaction, Toulouse, France). A beam of light passes through the emulsion, and it measures the transmission and the backscattering of the light. The sample is scanned from the bottom to the upper part of the cell and the figures represent a macroscopic fingerprint of the sample at a given time. The equipment can also calculate the Turbiscan Stability Index (TSI), which is a single value reflecting destabilization. The more unstable the sample is, the higher the value will be. Emulsions without lycopene were analyzed at 30 °C for 30 days, and those with lycopene for 10 days.

- Encapsulation efficiency

The EE is the percentage of lycopene that is encapsulated in oil droplets. It is defined by Equation (1):

(1) $$EE\left(\%\right)=\frac{{CL}_{total}-{CL}_{free}}{{CL}_{total}}\cdot 100$$

where CL_total_ is the total concentration of lycopene in the emulsion, understood as the theoretical value, 0.2 g/L, and CL_free_ is the free concentration, referred to the lycopene that remains not encapsulated.

The lycopene concentration of the samples was measured with a Genesys 150 UV spectrophotometer (Thermo Scientific, Waltham, MA, USA), using the absorbance value at 472 nm, according to the method reported by Zhao [43] with slight modifications. Briefly, 1 mL of emulsion was collected and dissolved in 1 mL of *n*-hexane, vortexed for 1 min at 1000 rpm and centrifugated for 10 min at 5000 rpm. The supernatant, which corresponds to the organic phase (*n*-hexane with lycopene dissolved), was collected and set aside. One mL of *n*-hexane was added to the aqueous phase, repeating the previous process (vortexed at 1000 rpm, 1 min followed by centrifugation at 5000 rpm, 1 min). The supernatant was collected, combined with the previous one and diluted 10 times with *n*-hexane. Emulsions without lycopene were used as a control. The spectrophotometer was calibrated with different concentrations of lycopene in hexane. The standard curve obtained was CL_free_ =7.2753·A_427_ (R^2^ = 0.9998).

To analyze lycopene stability, emulsions were stored in the absence of light at two different conditions, 5 and 20 °C. The previous procedure was repeated at several intervals of time for the stored emulsions.

Loading capacity (LC) was also calculated regarding the encapsulated lycopene on oil drops, taking into account the CL_free_ obtained from the calibration curve.

## 4. Conclusions

Stable soybean O/W emulsions obtained by sustainable formulations using several types of chitosans have been prepared. Full coverage of oil drops was easily raised by non-acidified chitosans and acidified ones with high viscosity values.

The presence of lycopene in emulsions did not affect drop size and stability. Lycopene was satisfactorily encapsulated in emulsions formulated with soybean oil and stabilized either with surfactant or chitosan reaching EE values up to ~70–75% and loading capacities of 0.15 mg/g.

Lycopene-loaded emulsions present high stability after 10 days of storage, without significant differences for emulsions stored at 5 or 20 °C.

It has been demonstrated that chitosan is a good alternative as a sustainable stabilizer to partially replace the traditional synthetic ingredients in O/W emulsions containing lycopene. The emulsions prepared in the present study can be potentially used as additives for enriched natural pharmaceutical or food formulations. The use of new biodegradable, renewable and biocompatible formulations will contribute to reduce the environmental impact of synthetic materials and their ingestion by humans, reducing their risk of suffering from potential pathologies.

## Figures and Tables

**Table 1:** Mean diameters and TSI values of formulations and different stabilizers subjected to stability tests for 30 days.

Stabilizer	D_[4;3]_ t = 0 (µm)	D_[4;3]_ t = 30 d (µm)	TSI t = 30 d
2% T80/S80 = 1/0	18.7 ± 0.7	14.5 ± 0.4	33 ± 3
5% T80/S80 = 1/0	17.4 ± 0.5	12.8 ± 0.5	27 ± 3
2% T80/S80 = 3/1	12.3 ± 3.9	7.0 ± 0.3	29 ± 3
5% T80/S80 = 3/1	4.3 ± 0.2	3.1 ± 0.1	16 ± 2
6 g/L Ch1	22.8 ± 7.5	162.6 ± 2.1	43 ± 4
6 g/L Ch2	18.0 ± 0.2	46.6 ± 0.2	45 ± 4
6 g/L Ch3	23.8 ± 0.4	39.9 ± 0.1	35 ± 3
6 g/L Ch4	10.7 ± 0.2	34.7 ± 0.3	50 ± 5
6 g/L Ch5	13.4 ± 0.3	59.2 ± 1.6	61 ± 5

**Table 2:** Mean diameters, TSI, loading capacity (LC) and lycopene stability (LS) values for the soybean oil emulsions with lycopene. All samples were stored in the absence of light.

Emulsifier	T_storage_ (°C)	D_[4;3]_ t = 0 (µm)	D_[4;3]_ t = 10 d (µm)	TSI t = 10 d	LC (mg/g) t = 0	LS (mg/g) t = 10 d
5% T80/S80 = 3/1	5	4.3 ± 0.3	4.9 ± 0.1	-	0.15 ± 0.02	0.15 ± 0.02
	20		4.0 ± 0.3	23.72 ± 3		0.14 ± 0.01
6 g/L Ch3	5	19.6 ± 2.8	23.1 ± 0.1	-	0.14 ± 0.01	0.15 ± 0.01
	20		21.7 ± 0.3	28.89 ± 3		0.08 ± 0.02
6 g/L Ch4	5	15.2 ± 2.1	17.3 ± 0.1	-	0.09 ± 0.02	0.08 ± 0.01
	20		24.6 ± 0.4	37.80 ± 4		0.07 ± 0.01

**Table 3:** Nomenclature used for the different types of chitosan.

Chitosan Viscosity (mPa·s)	Nomenclature	Chitosan Molecular Weight (kg/mol)	Nomenclature
5–20	Ch1	100–300	Ch4
20–100	Ch2	600–800	Ch5
200–600	Ch3		

## Data Availability

The raw data supporting the conclusions of this article will be made available by the authors on request.
